# Moving higher and higher: imitators’ movements are sensitive to observed trajectories regardless of action rationality

**DOI:** 10.1007/s00221-017-5006-4

**Published:** 2017-06-17

**Authors:** Paul A. G. Forbes, Antonia F. de C. Hamilton

**Affiliations:** Institute of Cognitive Neuroscience, University College London, London, UK

**Keywords:** Imitation, Motor contagion, Obstacle priming, Perception–action coupling

## Abstract

Humans sometimes perform actions which, at least superficially, appear suboptimal to the goal they are trying to achieve. Despite being able to identify these irrational actions from an early age, humans display a curious tendency to copy them. The current study recorded participants’ movements during an established imitation task and manipulated the rationality of the observed action in two ways. Participants observed videos of a model point to a series of targets with either a low, high or ‘superhigh’ trajectory either in the presence or absence of obstacles between her targets. The participants’ task was to watch which targets the model pointed to and then point to the same targets on the table in front of them. There were no obstacles between the participants’ targets. Firstly, we found that the peak height of participants’ movements between their targets was sensitive to the height of the model’s movements, even in the ‘superhigh’ condition where the model’s action was rated as irrational. Secondly, participants showed obstacle priming—the peak height of participants’ movements was higher after having observed the model move over obstacles to reach her targets, compared to when there were no obstacles between her targets. This suggests that participants code the environment of co-actors into their own motor programs, even when this compromises the efficiency of their own movements. We discuss the implications of our findings in terms of theories of imitation and obstacle priming.

## Introduction

Imagine you are at a wedding and the bride proposes a toast. You observe as she reaches over a coffee cup to pick up her glass. You then prepare to reach for your own glass. There is no cup obstructing your path to it. If human imitation is rational, your reach trajectory should not be perturbed by the recent observation of the bride’s obstacle avoidance (Gergely and Csibra 2003). Why move with a higher trajectory when there is no need to? Yet, increasing evidence suggests that when moving within a social context, humans often perform actions which, at least superficially, appear suboptimal to the goal they are trying to achieve.

The ability to identify these seemingly irrational actions may be present early in development (Gergely and Csibra 2003) and the observation of irrational, compared to rational, actions results in distinct patterns of neural activity (Brass et al. 2007; Marsh et al. 2014). Despite this competency in identifying irrational actions, humans display a curious tendency to both implicitly and explicitly copy them (McGuigan et al. 2011; Marsh et al. 2013; Griffiths and Tipper 2009; Hardwick and Edwards 2011).

The current study recorded participants’ movements during an established imitation task and manipulated the rationality of the observed action in two ways (Wild et al. 2012; Forbes et al. 2016). Firstly, we aimed to establish whether participants’ own movements would be sensitive to height of a model’s trajectory even when this was exaggeratedly high and rated as irrational. Secondly, we aimed to establish whether participants code the environment of the model into their own motor programs, even when this compromises the efficiency of their own movements.

### Motor contagion: observed actions influence the observer’s own actions

Action perception and action production are closely related (James 1890; Prinz 1997). This link has been robustly demonstrated behaviourally using a range of experimental approaches (see Krishnan-Barman et al. 2017; Becchio et al. 2010; for reviews). Automatic imitation studies have highlighted that the observation of a congruent action speeds the execution of a subsequent action (e.g. observe hand opening/perform hand opening), whereas the observation of an incongruent action slows the execution of a subsequent action (e.g. observe hand opening/perform hand closing) (Brass et al. 2000; see Heyes 2011 for a review). Motor interference tasks have shown that the execution of a continuous sinusoidal movement (e.g. in the horizontal plane) is perturbed by the observation of a similar movement in the orthogonal plane (e.g. in the vertical plane; Kilner et al. 2003). Similarly, imitation studies using motion tracking have shown that participants’ movements are sensitive to the kinematics of recently observed movements, such as the velocity and height of a model’s movements (Wild et al. 2010; Forbes et al. 2016).

These findings in combination with neurophysiological (Di Pellegrino et al. 1992) and neuroimaging data (Kilner et al. 2009; Oosterhof et al. 2013) suggest that our motor systems are readily influenced by the observation of another agent’s actions: a phenomenon known as motor contagion (Blakemore and Frith 2005). Whilst the extent of motor contagion appears to be influenced by a range of social cues (Wang and Hamilton 2012), such as the animacy (Liepelt and Brass 2010), group membership (Rauchbauer et al. 2015; van Schaik et al. 2016), and intention of the observed agent (Liepelt et al. 2008), whether the rationality of the observed action influences motor contagion remains relatively unexplored.

### Rationality and overimitation

The ability to identify and reason about the rationality of a goal-directed action may be present from approximately 12 months of age (Gergely and Csibra 2003; Kamewari et al. 2005; Scott and Baillargeon 2013; Sodian et al. 2004). In addition to this early competency in distinguishing rational from irrational actions, adult neuroimaging studies have revealed that the brain’s mentalising system, specifically the temporoparietal junction, medial prefrontal cortex (Marsh and Hamilton 2011) and superior temporal sulcus (Brass et al. 2007), distinguishes rational from irrational actions. Medial prefrontal cortex may be particularly attuned to unusual or irrational actions. Desmet and Brass (2015) showed that the observation of unusual *intentional* actions (e.g. an agent deliberately closes a box with her arm rather than her hand) was related to activation in the anterior medial prefrontal cortex, whilst the observation of unusual *accidental* actions (e.g. an agent bumps her arm against a box and closes it) was associated with activation in dorsal and posterior parts of medial prefrontal cortex (Desmet and Brass 2015).

Given our early competency in identifying irrational actions and the brain’s sensitivity to these types of actions, human’s tendency to overimitate, that is, copy seemingly arbitrary and unnecessary features of an action, seems somewhat peculiar (McGuigan et al. 2011). During a typical overimitation task, participants observe a model perform a causally irrelevant action, such as tapping on top of a box, and subsequently copy this action when given the object. This is despite participants rating the action as “silly,” so not required to complete the end-goal of the action, such as retrieving a toy from the box (Marsh et al. 2013). Overimitation may be present from approximately 18 months (Nielsen 2006), shortly after infants show sensitivity to irrational actions (Gergely and Csibra 2003). The tendency to overimitate then increases with age during childhood and persists into adulthood (McGuigan et al. 2011; Whiten et al. 2016). Whiten et al. (2009) have tried to explain overimitation in terms of a “copy all, refine later” strategy. Whilst in some circumstances (e.g. during overimitation tasks) this may result in the imitator performing inefficient actions, in general such a strategy may be adaptive as objects and tasks are often “casually opaque” (Lyons et al. 2007). For example, it is often not immediately clear how a novel object functions or what the particular requirements of a task are.

Overimitation tasks usually involve participants explicitly copying an unnecessary action performed on an object. However, motor contagion studies have shown that participants’ own movements are also sensitive to unnecessary and task irrelevant aspects of observed movement trajectories (Griffiths and Tipper 2009; Hardwick and Edwards 2011; Wild et al. 2010) For example, Hardwick and Edwards (2011) asked participants to perform reaching and grasping actions to an object after having observed, or whilst observing, an experimenter reaching with a normal, or an exaggeratedly high, trajectory. Despite being instructed to perform normal reaching movements throughout the experiment, participants’ maximum wrist height was approximately 3 mm higher after having observed, or whilst observing, the experimenter reach with an exaggeratedly high trajectory. Hardwick and Edwards concluded that even exaggerated movement kinematics have a small but significant effect on the observers’ own movements.

Furthermore, several studies have demonstrated that people imitate in strategic games even when this impairs performance. For example, Cook et al. (2012) asked people to play rock–paper–scissors either with or without a blindfold (Cook et al. 2012). When both players wore blindfolds, the number of draws (i.e. when both participants made the same gesture) was at chance. However, when only one player was blindfolded, the frequency of draws was elevated. This was despite participants being instructed to win as much as possible. This tendency to imitate even when it compromises people’s performance and financial payoffs has further been demonstrated in a variant of the whac-a-mole arcade game (Naber et al. 2013) and in players of matching pennies (Belot et al. 2013).

### ‘Irrational’ movements: learning and communicating

So what could be behind this tendency to copy and be influenced by irrational movements? Gergely and Csibra (2006) argued that during development ostensive pedagogical cues are vital in triggering and facilitating imitative learning (Gergely and Csibra 2006). Infants adopt a ‘pedagogical stance’ whereby they attend to and are influenced by exaggerated (and seemingly irrational) movements. Brand et al. (2002) showed that caregivers display “motionese”—they enhance or exaggerate features within an action sequence to facilitate the infant’s processing of that action. For example, a slow or curved trajectory towards a target can make the goal or intention of an action more salient. Nagai and Rohlfing (2007) argued that “motionese” can help infants to determine what to imitate. Using a saliency-based attention model, which was sensitive to the colour, intensity, orientation, flicker, and motion of the visual scene, they found that motionese could also be utilised by robots when determining what to imitate—even in the absence of existing knowledge about task-related actions or the action goals.

Whilst exaggerated movement trajectories are important for imitation and learning during development, more generally they are important for “joint action optimization” (Pezzulo et al. 2013). Pezzulo et al. proposed that during joint actions co-actors move in such a way as to optimise the success of the interaction (e.g. moving a sofa) rather than their own movements within that interaction (e.g. moving their half of the sofa). This could mean changing one’s own movements—and inferring a cost—to benefit the joint action. For example, an exaggerated trajectory requires more effort and potentially a more awkward position but if it signals to a co-actor which direction one is moving the sofa, then this optimises the success of the joint goal. This theory was supported by Vesper et al. (2016) who showed that when pairs of participants were required to arrive at a target at the same time, they exaggerated the curvature of their movements to communicate their arrival time.

Given the importance of exaggerated movement trajectories for learning and their role in joint action optimisation, we aimed to test whether participants’ own movements would continue to be sensitive to exaggerated and irrational movement trajectories even when this compromised the efficiency of their own movements. Alternatively, a mechanism could exist which evaluates the rationality of the observed movement trajectory so that participants’ own movements cease to be influenced by them. This could be similar to the mechanisms by which other ‘top down’ factors modulate imitative behaviours, such as the presence of goals (Wild et al. 2010) and the range of social cues outlined above (Wang and Hamilton 2012). In the brain, this ‘top-down’ control of imitative behaviours by social cues, such as eye contact, has been shown to be modulated by medial prefrontal cortex (Wang et al. 2011). Given that medial prefrontal cortex responds to action rationality (Desmet and Brass 2015; Marsh and Hamilton 2011), it is plausible that a similar mechanism may also modulate the imitation of irrational movement trajectories.

### Coding the environment of others into our own

When we move within a social context, we often encode our own environment in terms of other peoples’ points of view. For example, Frischen, Loach and Tipper (2009) asked participants to reach to a target in the presence of a distracter. In such tasks, participants display negative priming—after having moved to the target, responses to the distracter (i.e. the previously ignored stimulus) are slowed (Tipper 1985). This is due to participants initially inhibiting responses to the distracter. Frischen et al. (2009) found that when participants performed the task by themselves, they displayed an egocentric frame of reference—negative priming was strongest for distracters closest to their hand compared to those further from it. In contrast, when participants took turns to complete this task with another person, participants displayed an allocentric frame of reference—negative priming was strongest for distracters that were salient for the other person rather than themselves. They concluded that “observers are essentially processing their environment in the same way that the *other person* is encoding it as *they* are interacting with their surrounding” (Frischen et al. 2009; p. 218).

The finding that we may code the environments of others in our own action space was supported by Griffiths and Tipper (2009) who asked participants to reach for and lift up a target block in the presence or absence of an obstacle. On trials where there was no obstacle, but in the previous trial they had moved over an obstacle, participants’ reach trajectory was higher compared to trials where the previous trial contained no obstacle. When participants took turns to complete the task with another participant, they found that the obstacle avoidance of one participant influenced the reach trajectory of the other but only if the observed obstacle was in the peripersonal action space of the participant. If the obstacle was outside “the comfortable reach space of the observer”, this had no effect on reach trajectory. However, this view was later challenged by Griffiths and Tipper (2012) who found that the observed obstacle avoidance could take place outside of the peripersonal space of the participant and induce obstacle priming, but this was dependent on participants sharing their workspace with their co-actor and having a sense of ‘shared ownership’ over it. In their ‘shared workspace’ condition, there was just one set of objects which the experimenter moved between the two participants after each trial. In contrast, when the participants had separate workspaces, so one participant had a yellow obstacle and target and the other had a blue set; obstacle priming did not occur.

Further constraints on obstacle priming have been outlined by Roberts et al. (2016). Participants watched videos of an actor perform horizontal or curvilinear sinusoidal movements either in the presence or absence of a cylindrical object. Participants’ task was to perform continuous horizontal arm movements. The object in the video acted as either an obstacle, so required the actor in the video to move with a particular trajectory to avoid it, or as a distracter, so was present in the video but was not in the path of the actor’s trajectory. As previously shown (Roberts et al. 2014), participants’ movement deviation in the vertical plane was greater when observing curvilinear compared to horizontal movements. When observing horizontal movements, however, deviation increased in the presence of an obstacle. Conversely, movement deviation was not modulated by the presence of an obstacle or distractor in the curvilinear condition. Roberts et al. (2016) proposed that the observed environmental context, such as the presence of an obstacle, only influences participants’ own movements when the observed and executed actions are congruent (i.e. both actor and participant were performing horizontal movements).

The mechanism behind obstacle priming was recently explored by van der Wel and Fu (2015) who aimed to investigate whether obstacle priming was the result of entrainment or co-representation of the model’s action (van der Wel and Fu 2015). Entrainment refers to the unintentional, rhythmical synchronisation of two individuals’ actions. For example, two people sat observing each other in rocking chairs tend rock together in synchrony (Knoblich and Sebanz 2008; Richardson et al. 2005). Whereas, co-representation involves symbolically representing the goals and intentions of the co-actor (Sebanz et al. 2005). Van der Wel and Fu (2015) proposed that the mechanism may vary depending on the nature of the action.

They asked participants to move a dowel between two locations to the pace of a metronome. The metronome produced either a continuous sequence (a continuous looping of a tone every 850 ms) or a discrete sequence (two tones separated by 850 ms with a pause following each set). This ensured that participants produced either discrete or continuous movements with the dowel. Participants sat next to a confederate who moved his own dowel to a discrete or continuous sequence between his own two targets, whilst the participant was performing the same movement. On some trials, the confederate had an obstacle between his targets and on other trials there was no obstacle. The key manipulation was whether participants could see the action of the confederate. For discrete movements, obstacle priming—the difference between the participants’ peak height when the there was an obstacle between the confederate’s targets compared to when there was no obstacle—was the same regardless of whether participants could see the actions of the confederate or not. For continuous movements, however, obstacle priming only occurred when participants could see the action of the confederate. Van der Wel and Fu (2015) argued that obstacle priming during continuous movements is due to entrainment and, thus, dependent on visual information; whilst during discrete movements, obstacle priming is driven by co-representation of the actor’s task.

### Current aims

#### Imitation

The first aim of Experiment 1 was to test the limits of participants’ sensitivity to irrational movement trajectories. Our previous work and that of others has demonstrated that participants’ own movements are sensitive to “high” and “low” (Forbes et al. 2016; Griffiths and Tipper 2009; Hardwick and Edwards 2011) and “fast” and “slow” observed movement trajectories (Wild et al. 2010). However, in these studies the manipulation of height and speed was relatively subtle. For example, the difference between the high and low trajectory in Hardwick and Edwards (2011) was 7 cm, and 8 cm in Forbes et al. (2016). Our aim was to establish whether participants’ movements would continue to be sensitive to the height of the model’s movement trajectory even when these were clearly exaggerated and rated as irrational. As before the height of the actor’s trajectory was manipulated, there was a low and high condition (Forbes et al. 2016), but we also included an additional “superhigh” condition. The peak height of the model’s trajectory in the superhigh condition was 12 cm greater than that in the low condition. We aimed to test whether participants’ own movements would continue to be sensitive to the trajectory in this superhigh condition. Experiment 2 was conducted to obtain rationality ratings of the movement trajectories to test whether the superhigh movements were deemed more irrational than the high and low movement trajectories.

#### Obstacle priming

Experiment 1 also aimed to build on the obstacle priming literature in several ways. Firstly, in Roberts et al. (2016), participants were required to make a pre-specified, continuous, horizontal movement so the congruency between the observed and executed movement was determined by the experimental condition (i.e. whether they were observing horizontal or curvilinear movements). In the current study, participants observed videos of an actor pointing to a series of three targets out of a four possible targets. The actor in the video moved with a low, high or ‘superhigh’ (i.e. exaggeratedly high) trajectory either in the presence of absence of obstacles between her targets. The participants’ task was to point to the same targets the actor pointed but there were no obstacles between the participants’ targets. So, rather than being instructed to make a pre-specified movement, participants were given goal-orientated instructions (i.e. “point to the same targets she pointed to”) and were free to point to these targets with a trajectory of their choosing. Here, much like during the toast at the wedding, the goal was pre-determined (i.e. pick up the glass/point to the targets), yet participants could decide for themselves how they achieved this goal. Thus, our aim was to establish whether participants would be influenced by the observed obstacle even when the exact nature of the required movement was not explicitly pre-specified.

Our second aim concerned the location of model’s obstacle. Griffiths and Tipper (2009, 2012) suggested that the observed obstacle needed to be within “the comfortable reach space of the observer” (i.e. their peripersonal action space) to have an effect on their movements (Griffiths and Tipper 2009), or participants must feel they are sharing their workspace with their co-actor (Griffiths and Tipper 2012). In our task, participants sat 70 cm from a screen which displayed videos of an actor moving over obstacles to point to a series of targets. This ensured that the obstacles were outside of the participant’s peripersonal action space. Moreover, there was a clear divide between the workspace of the participant which was on the table in front of them and that of the actor whose workspace appeared on a screen in front of them. Whereas Griffiths and Tipper (2009, 2012) required participants to pick up a target block, we aimed to investigate obstacle priming within an imitative pointing paradigm (Forbes et al. 2016; Wild et al. 2010, 2012). We investigated whether obstacle priming would occur even when the obstacle was outside of the participants’ peripersonal action space, and, when participants did not share a workspace with their co-actor. Finally, to ensure that any effects were not simply due to the visual saliency of the observed obstacle on the screen in front of the participants, we included a condition where the actor’s obstacle was half the size.

## Experiment 1

### Methods

#### Participants

Participants (*N* = 27, three male) were obtained from the UCL Institute of Cognitive Neuroscience participant database and had a mean (±SD) age of 22.0 (±2.9) years. All participants were right-handed, had normal or corrected-to-normal vision and reported no history of neurological disorder. All were financially reimbursed for their time and gave written informed consent before participating. All procedures were approved by the UCL Research Ethics Committee.

#### Stimuli

**Fig. 1:**
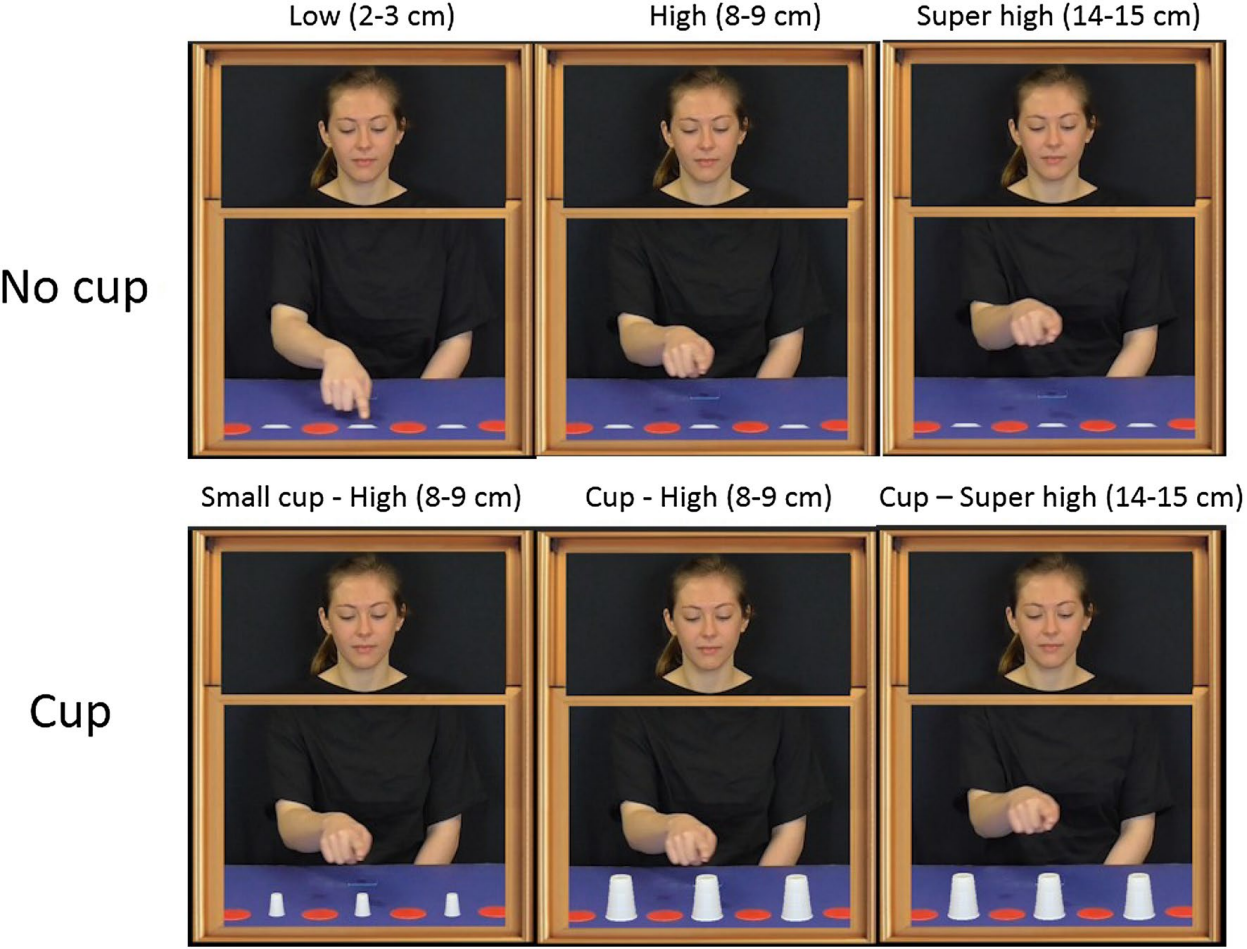
The six conditions; peak height for each condition is shown in* parentheses*

Videos consisted of a female actor positioned behind a window frame, which appeared 48 cm × 40 cm on the projector screen. There was a horizontal bar across just above the centre of the window frame so the screen could be split into two (see Fig. 1). The top half of every video was the same for all trials and started with the actor looking up and smiling before looking down at her hand. At this point in the bottom half of the video, the actor moved from her resting position and pointed to three out of a possible four targets on the table in front of her in sequence. She then returned to the resting pad. Each target was a red circle, which appeared 4 cm in diameter, and the centre of the targets appeared 10.5 cm away from each other on the projector screen. There were eight different movement combinations.

There were six different conditions (see Fig. 1): three height conditions (low, high, superhigh) and three cup conditions (high small cup, high cup, superhigh cup). In the height conditions, the actor moved above the table with a peak height of approximately 2–3 cm between the targets for the low condition, 8–9 cm for the high condition and 14–15 cm for the superhigh condition. The initial movement to the first target and the fourth (final) movement back to the resting pad were also manipulated to be either low, high or superhigh.

Between the targets in the height conditions, there was a flat, white marker (4 cm diameter). There were three cup conditions. These used the same videos as for the high and superhigh videos except a cup (4 cm × 5.50 cm) was superimposed between the targets for the high cup condition and superhigh cup condition. The high small cup condition used the high condition videos but the cup superimposed between the targets was half the size of the cups used in the other cup conditions. Videos were edited using Adobe Premier Pro CC 2015 (Adobe systems, San Jose, CA, USA) and presented using Vizard (WorldViz Inc, Santa Barbara, USA).

#### Procedure

**Fig. 2:**
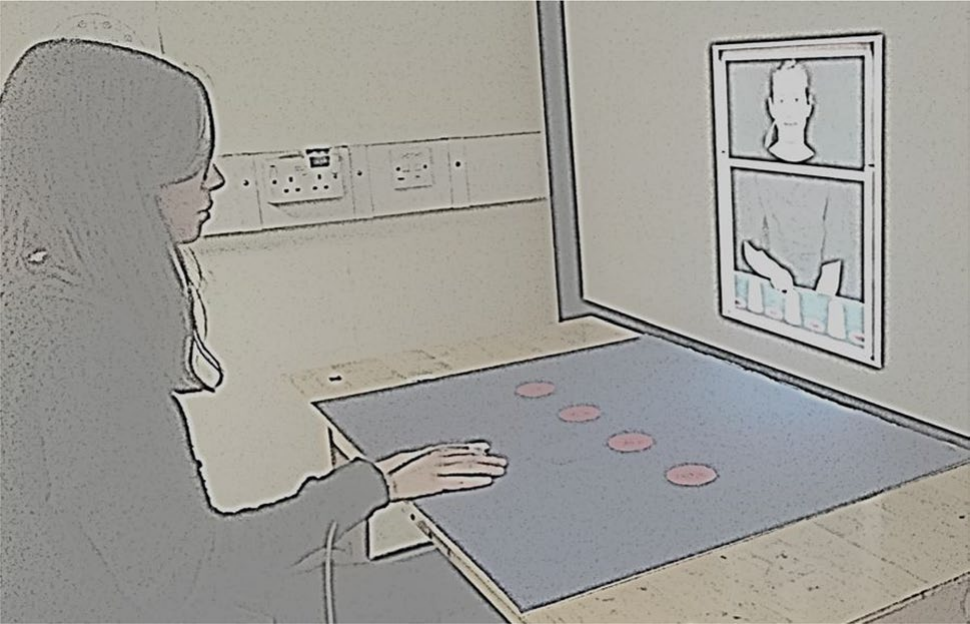
The experimental setup

Participants sat approximately 70 cm from the projector screen (Fig. 2). An electromagnetic marker (Polhemus LIBERTY system, Colchester, USA) was attached to participants’ right index finger which enabled finger movements to be recorded. On the table in front of the participants, there was a piece of 81 cm × 66 cm blue card with four 6 cm diameter red circles stuck in the middle of it. The centre of the circles was 15 cm apart from each other and was 30 cm in front of the participants. These red circles acted at the targets. There was also a 6 cm × 4 cm piece of blue card stuck 10 cm in front of the participant which acted at the ‘resting pad’ where participants were required to place their right index finger when not moving. There were no cups in front of the participants.

Before the start of the experiment participants completed calibration: they placed their right index finger into the middle of each of the four targets and the resting pad so that the coordinates could be recorded. After calibration participants were given eight practice trials. All the practice videos contained no cups. Participants were instructed to rest their finger on the resting pad in front of them. They were told to watch the actor in the video and see which three targets she points to, then, when she returns to her resting position, they should point to the same targets she pointed to in the same order. The spatial correspondence between the targets in the video and the targets in front of the participants was explained. For example, participants were told that if the actor in the video pointed to the target on ***her*** far left then participants should point to the target on their far right so that there was a spatial match between their targets. After practice, participants completed three identical blocks with 48 trials in each block (6 conditions × 8 different target combinations). Videos were presented in a randomised order.

**Table 1:** Mean and SD for each of the six conditions

Peak height (cm)	No cup low	No cup high	No cup superhigh	Small cup high	Cup high	Cup superhigh
Mean	3.82	4.96	5.40	5.14	5.12	5.89
SD	1.42	2.10	2.80	2.30	2.40	3.09

### Results

#### Excluded data

The movement data were analysed using Matlab R2013b (MathsWorks, Natick, USA) and filtered with a Butterworth filter to remove high frequencies. For each trial, each of the participant’s data was chunked into four movements using their calibration file: (1) the movement to the first target from the resting pad, (2) the movement to the second target; (3) the movement to the third target, and (4) the movement back to the resting pad. Three participants were excluded from the final analysis as over 10% of their trials could not be chunked correctly (error rates: 25.5, 17.7 and 14.6%). The error rates for the other participants were all below 10% (mean 1.8%, SD 2.2%).

#### Peak height analysis

Mean peak height of the movements between the targets (mean of movements 2 and 3) for each condition for each participant was subject to repeated measures ANOVAs. The means and standard deviations for each condition are shown in Table 1.

#### Height

**Fig. 3:**
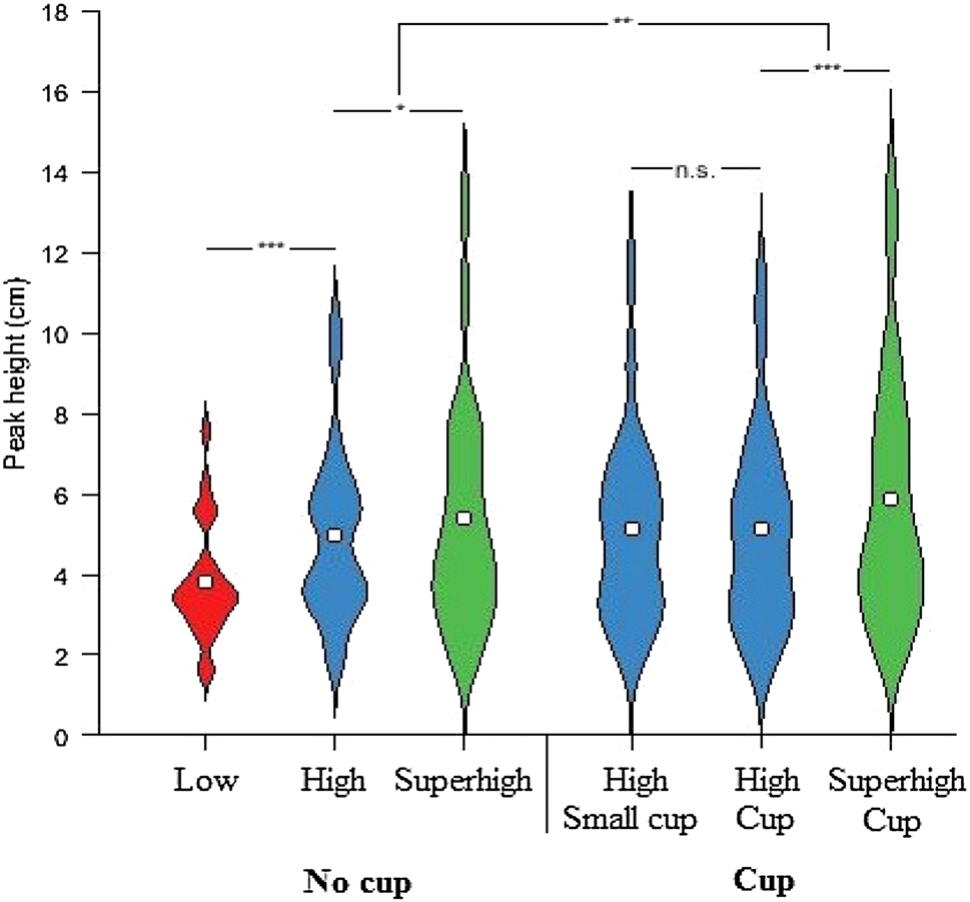
Mean peak height between the targets

A one-way, repeated measures ANOVA was conducted with height as a factor for the three no cup conditions (low, high, superhigh). Epsilon (ε) = 0.597 as calculated to Greenhouse and Geisser (1959) was used to correct the one-way ANOVA. This revealed a main effect of height (*F*_1.194, 27.457_ = 12.09, *p* = 0.001, *η*_p_^2^ = 0.344; Fig. 3). Post hoc paired samples t tests revealed that the peak height of participants’ movements was significantly higher in the high condition [mean (SD): 4.96 cm (2.10)] compared to low condition [mean (SD): 3.82 cm (1.42)], (*t*_23_ = 3.824, *p* < 0.001, *d* = 0.781), and significantly higher in the superhigh condition [mean (SD): 5.40 cm (2.80)] compared to the high condition (*t*_23_ = 2.080, *p* = 0.049, *d* = 0.425).

#### Cup

A 2 × 2 repeated measured ANOVA was conducted with height (high/superhigh) and cup (cup/no cup) as factors. This revealed a main effect of cup (*F*_1, 23_ = 9.325, *p* = 0.006, *η*_p_^2^ = 0.288) with participants displaying a greater peak height in the cup conditions [mean (SD): 5.50 cm (2.73)] compared to the no cup conditions [5.18 cm (2.42)], and also a main effect of height (*F*_1, 23_ = 13.189, *p* = 0.001, *η*_p_^2^ = 0.364) with participants displaying a greater peak height for the superhigh compared to high conditions (Fig. 3). There was no significant interaction between cup and height (*F*_1, 23_ = 2.543, *p* = 0.124, *η*_p_^2^ = 0.100). Finally, there was no significant difference between the high cup and high small cup condition as shown by paired samples *t* test (*t*_23_ = 0.350, *p* = 0.730, *d* = 0.071).

## Experiment 2

Experiment 2 was conducted to obtain rationality ratings of the movements in the low, high and superhigh conditions.

### Methods

#### Participants

One hundred participants (30 female, 3 left-handed) with a mean age of 27 years (range 18–54) were recruited via the Prolific Academic website (http://prolific.ac). The study took approximately 5 min to complete and participants received £0.50 in exchange for their participation. Ethical approval was granted by the UCL Research Ethics Committee and informed consent was obtained from all participants.

#### Stimuli and procedure

An example video for each of the three height conditions (low, high, superhigh) was shown to the participants. The same movement combination was used for all three height conditions. Participants showed each video three times and asked to rate the rationality of the action in the video, using a battery of three statements (adapted from Marsh et al. 2014). The statements were: (1) ‘This action seems unnatural’, (2) ‘The action seems efficient’ and (3) ‘I would complete this action differently.’ Participants were asked to watch the action and then indicate how much they agreed or disagreed with each statement on a 5-point scale. This created a total of nine trials. The scores on these statements were summed, with the scores on Statement 2 reversed scored, to produce an aggregated irrationality rating (with a maximum score of 15) for each of the three height conditions. The experiment can be seen here: testable.org/t/81590f313.

### Results

#### Excluded data

If participants response time was less than 5 s for at least one of the nine trials, then they responded before the end of the action in the video and their data were excluded from the analysis (*n* = 19). Similarly, if participants’ response times were greater than 60 s for at least one of the nine trials, they were excluded from the analysis as it is likely they became distracted during the trial (*n* = 3). One participant showed response times both shorter than 5 s and greater than 60 s, so the final sample consisted of 79 participants (25 female, 2 left-handed) with a mean age of 28 years (range 18–54).

#### Irrationality rating

**Fig. 4:**
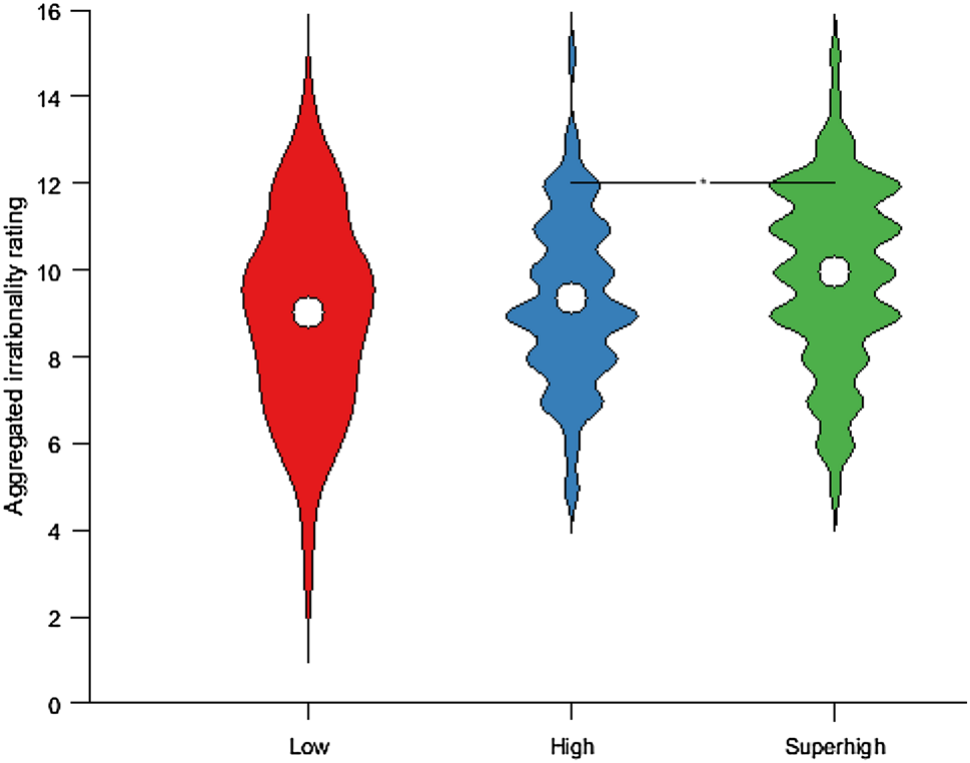
Mean aggregated irrationality ratings for the movements in the three conditions

The aggregated irrationality ratings were subject to a one-way repeated measures ANOVA with height (low, high, superhigh) as a factor. Epsilon (*ε*) = 0.882 as calculated to Greenhouse and Geisser (1959) was used to correct the ANOVA. This revealed a main effect of height (*F*_1.76, 137.55_ = 4.389, *p* = 0.018, *η*_p_^2^ = 0.053; Fig. 4). Post hoc paired samples t tests revealed that the irrationality ratings were significantly greater (*t*_78_ = 2.347, *p* = 0.021, *d* = 0.264) for videos showing the superhigh movements [mean (SD): 9.95 (2.06)] compared to those showing high movements [mean (SD): 9.34 (1.91)]. There was no significant difference between the irrationality ratings for the high [mean (SD): 9.34 (1.91)] and low [mean (SD): 9.04 (2.27)] movement videos (*t*_78_ = 0.970, *p* = 0.335, *d* = 0.109).

## Discussion

The current study used an established sequential pointing task to determine whether the rationality of an observed movement trajectory influenced the extent to which participants’ movements were influenced by it. Firstly, we found that participants’ pointing movements between a series of targets were sensitive to the height of an actor’s movement trajectory (Experiment 1), even when the observed movement trajectory was rated as irrational (Experiment 2). Secondly, we examined participants’ movements after having observed an actor move over obstacles to reach her targets. Participants moved with an even higher trajectory between their own targets after having observed these videos, compared to videos in which there were no obstacles between the actor’s targets. This was despite there being no obstacle between the participants’ own targets throughout the experiment. This suggests that participants’ movements are not only influenced by the observed movement but also the environment within which the observed movement took place. We discuss the implications of our findings in terms of theories of imitation and obstacle priming.

### Moving higher and higher

Our findings replicate previous work demonstrating that participants’ own movements are sensitive to the height of a recently observed movement trajectory (Forbes et al. 2016; Griffiths and Tipper 2009; Hardwick and Edwards 2011; Wild et al. 2010). They also extend this work in several important ways. In previous studies, the difference between the high and low observed movement trajectories was relatively subtle (Forbes et al. 2016; Hardwick and Edwards 2011). However, in the present study we showed that participants’ movements continue to be sensitive to movement trajectories which are clearly exaggerated and rated as irrational (i.e. the superhigh condition). It is not clear from our findings whether participants sensitivity to the superhigh condition was a form of motor contagion, similar to that caused by any biological motion (Blakemore and Frith 2005), or whether this is a type of overimitation whereby participants are aware that the exaggerated trajectory is causally irrelevant yet still explicitly copy it (e.g. McGuigan et al. 2011). There was considerable variation in the performance of our participants in the superhigh condition, so it is possible that both mechanisms were operating. More generally, our findings support work highlighting humans’ sensitivity to exaggerated movements trajectories (Brand et al. 2002; Desmet and Brass 2015). It is likely that the pedagogical (Gergely and Csibra 2006) and communicative value (Pezzulo et al. 2013) of exaggerated movement trajectories could be driving participants’ tendency to be influenced by them.

It is important to note that whilst the vast majority of participants moved higher in the high compared to low condition, fewer distinguished between the high and superhigh condition (although the difference in peak height reached statistical significance). Indeed, a minority of participants moved with a lower trajectory in the superhigh condition compared to the high condition. It remains to be seen what factors predict a breakdown of motor contagion (and/or overimitation) following the observation of exaggerated movement trajectories. For example, is this due to a deliberate evaluation of action rationality and/or is this related to people’s propensity to overimitate? Forbes et al. (2016) showed that whilst the movements of autistic adults’ are sensitive to the height of observed actions, this effect is reduced compared to neurotypical participants. Moreover, overimitation studies suggest that autistic children are less likely to copy task irrelevant actions (Marsh et al. 2013). Future studies should explore whether autistic traits predict a breakdown of motor contagion in the superhigh condition and, if so, why.

Hardwick and Edwards (2011) required participants to make simple, overlearned reach-to-grasp movements towards an object. They proposed that participants’ sensitivity to high movement trajectories during this task is in line with the goal-directed theory of imitation (Bekkering et al. 2000). This theory states that imitators breakdown an observed movement into a hierarchy of goals, whereby goals of greater importance (e.g. pick up the cup) are imitated more readily than those deemed of lesser importance (e.g. pick up the cup by its handle). When the task is simple, such as during reach-to-grasp actions, observers have the cognitive resources to attend to and copy multiple goals within the goal hierarchy, for example, both the outcome and the kinematics of the observed movement. Conversely, if cognitive resources are limited, for example during early childhood or when the task is more demanding, imitators prioritise goals further up the goal hierarchy (Bekkering et al. 2000). Similarly, if a goal is made particularly salient, then participants will imitate this goal more readily than goals further down the hierarchy (Wild et al. 2010).

Our task required participants to remember the sequence of the three targets the actor pointed and then point to their own targets in the same order. Our task was therefore more demanding than that of Hardwick and Edwards (2011). Despite this increased demand, participants’ own movements were still sensitive to the peak height of the actor’s movements. It is possible that the saliency of the movement trajectory resulted in participants’ movements being sensitive to it, despite the increased cognitive load. Future studies should directly manipulate the saliency of the elevated trajectory and the task demands, for example, by having participants point to more targets, to directly test the goal-directed theory of imitation within this sequential pointing paradigm.

### Coding the environment of others into our own

The present findings are in line with previous work demonstrating that participants code the environment of the observed model, such as an obstacle in her action space, into their own action space (Frischen et al. 2009; Griffiths and Tipper 2009, 2012; Roberts et al. 2016). Again, we build on this previous work in several important ways. Roberts et al. (2016) required participants to make a pre-specified, continuous, horizontal movement whilst observing a model make either a congruent (horizontal) or incongruent (curvilinear) movement. The presence of an obstacle in the video only influenced participants’ own movements when both the model and participant were performing the same horizontal movements. Whilst the goal of the observed and executed movement was congruent in our study (i.e. “point to the same targets that she does”), participants were less constrained in terms of the nature of the movement they were required to perform. That is, the trajectory of their movements was not explicitly pre-specified as it was in Roberts et al. (2016). This suggests that even when there is no direct matching between the observed and executed movement (i.e. they are not completely congruent), the environment of the model (i.e. the presence of obstacles) continues to influence participants’ own movements.

Secondly, Griffiths and Tipper (2009) suggested when we view another person avoiding an obstacle to reach for a target object, for this obstacle to influence our own reaching movement, it must be within our peripersonal action space. In their study, when the obstacle was beyond the “comfortable reach space” of the participant their reach trajectory was not perturbed by the obstacle. In our study, however, the obstacles between the actor’s targets were displayed on a screen 70 cm in front of the participants. Thus, they were outside of the peripersonal space of the participants. Despite this, participants’ movements between their own targets were higher after having observed the actor reach over obstacles to point to her targets, compared to when she moved with the same trajectory but there were no obstacles between her targets. This supports other work demonstrating that the proximity of the co-actor’s obstacle does not influence obstacle priming (van der Wel and Fu 2015).

Griffiths and Tipper (2012) later proposed that for obstacle priming to occur participants must share ownership of the workspace with a co-actor, even if the observed workspace is not within the peripersonal space of the participant. In our study, there was a clear divide between the workspace of the participant (i.e. the table they were sat at) and the actor’s workspace which was projected as a video onto a screen in front of the participants. Despite this separation between the workspaces, obstacle priming still occurred.

One possibility, however, is that due to the visual similarity between the workspaces (i.e. workspace in the videos looked like the workspace on the table in front of participants), participants may have felt they were sharing the workspace with the actor. Griffiths and Tipper (2012) created a sense of ‘separatedness’ between the two workspaces by having one participant interact with a blue set of objects and the other interact with a yellow set. In the shared workspace condition, participants interacted with the same workspace—after each trial, the workspace was moved across the table from one participant to the other by the experimenter. When interpreted in the light of our current findings, it is possible that the visual similarity between the observed workspace and the participant’s own workspace in Griffiths and Tipper (2012) may have been sufficient to cause obstacle priming, although it remains unclear why this was not the case in Griffiths and Tipper (2009; Experiment 2).

It should also be stressed that in Griffiths and Tipper (2009, 2012) the observed action was not relevant to the participant’s own subsequent action. Participants were instructed to passively observe the other person’s action. This is in contrast to the present study where participants were explicitly instructed to attend to the sequence of targets the model pointed to and then point to the same targets on the table in front of them. Thus, the present study was more similar to a joint action task (Sebanz et al. 2006), whereby the action of participants (i.e. the targets they pointed to) was dependent on the recently observed movements of the model. This greater attention to the model’s movements (Bek et al. 2016) and greater relevance of her movements to the participant’s task may also account for some of the differences in obstacle priming between the present study and those of Griffiths and Tipper (2009, 2012).

Van der Wel and Fu (2015) proposed that for discrete movements obstacle priming is due to the co-representation of the actor’s task (Sebanz et al. 2005). Conversely, for continuous movements obstacle priming is the result of entrainment and thus dependent on receiving concurrent visual information from a co-actor (Richardson et al. 2005). Our task required the execution of discrete movements after the participants had observed the actor move to her targets. So when participants pointed to their own targets, this was in the absence of concurrent visual information about the actor’s movements. Thus, our findings are generally in line with van der Wel and Fu’s (2015) interpretation that when performing discrete movements participants co-represent the task of the co-actor during obstacle priming. However, a co-representation account fails to fully account for our findings. According to a co-representation account, a smaller obstacle requires less adjustment yet there was no significant difference between the cup and small cup condition. Future studies should vary the size of the obstacle more systematically to directly test the co-representational account.

One possibility is that the continued presence of the obstacles on the screen in front of participants during their response period caused distractor interference (Tipper et al. 1997). However, we suggest that this interpretation is unlikely. Firstly, the obstacles were small (5.5 cm × 4 cm) and appeared 70 cm from the participants so well outside of their peripersonal action space. Secondly, as highlighted above, the saliency of the distractor has been shown to impact the extent of distractor interference (Tipper et al. 1998), yet when the size of the obstacle was halved (small cup condition) this did not impact the extent of obstacle priming. In addition, whilst automatic imitation of movement trajectories is at least partially due to spatial effects (i.e. the observed movements being higher up in space; Hardwick and Edwards 2012), the higher movements in the cup compared to the no cup conditions speak against a purely spatial effect. Here, the movements in the videos were same height, yet participants moved higher in the cup condition. If the effects were purely spatial, we would not expect to see these differences.

Finally, a potential avenue for future research could be to explore the effects obstacle avoidance has beyond participants’ reach trajectory. For example, Hayes et al. (2008) found that when participants observed videos of a model moving an object to avoid an obstacle, their affective ratings of the moved object were lower compared to when she moved the object without having to avoid an obstacle (Hayes et al. 2008). Hayes et al. (2008) argued that observing fluent actions, those in which the model does not need to avoid an object, results in more positive affective responses. It would be of interest to examine whether these affective effects of observing fluent actions also transfer to the model performing the action and the subsequent imitation of her actions.

## Conclusions

To conclude, we found that participants’ movements between a series of targets were sensitive to the height of observed movement trajectories, even when these were irrational—unnecessarily and exaggeratedly high. Secondly, the presence of obstacles between the model’s targets resulted in participants moving with an even higher trajectory between their own targets, despite there being no obstacles between them. This obstacle priming suggests that participants code the environment of a co-actor into their own action space. Our results are consistent with previous work demonstrating that obstacle priming is not dependent on the obstacles being within the peripersonal space of the participant (Griffiths and Tipper 2012). Our results also suggest that obstacle priming during the observation and execution of discrete movements is likely to depend on participants co-representing the task of the model (van der Wel and Fu 2015). The current paradigm provides a versatile platform to directly test this co-representational account and also explore other theories of imitation and obstacle priming.
